# Noninvasive mapping of shear strain predicts the anatomical distribution of mild traumatic brain injury^☆^

**DOI:** 10.1016/j.nicl.2026.103974

**Published:** 2026-02-19

**Authors:** Adnan A. Hirad, Doran Mix, Arun Venkataraman, Steven P. Meyers, Bradford Z. Mahon

**Affiliations:** a Department of Surgery, University of Rochester Medical Center, Rochester, NY 1462, USA; b Department of Neuroscience, University of Rochester Medical Center, Rochester, NY 14642, USA; c Del Monte Neuroscience Institute, University of Rochester, NY, USA; d Department of Biomedical Engineering, University of Rochester Medical Center, Rochester, NY 1462, USA; e Department of Physics and Astronomy, University of Rochester, NY 14623, USA; f Department of Imaging Sciences, University of Rochester Medical Center, Rochester, NY 1462, USA; g Department of Neurosurgery, University of Rochester Medical Center, Rochester, NY 1462, USA; h Department of Psychology, Carnegie Mellon University, Pittsburgh, PA 15206, USA; i Neuroscience Institute, Carnegie Mellon University, Pittsburgh, PA 15206, USA

## Abstract

Shear strain has been implicated as a key factor driving tissue injury in traumatic brain injury (TBI) (Holbourn, 1943, Ommaya et al., 1994, Margulies et al., 1990), but evidence has been lacking that links *in vivo* measures of strain in the human brain to the distribution of structural damage observed in TBI. In this study, we investigated the relation between strain concentration, measured using Magnetic Resonance Elastography (MRE), and changes in structural integrity, measured using Apparent Fiber Density (AFD), in mild TBI (mTBI) patients. We use MRE to show that regions commonly associated with differential brain tissue damage – such as the midbrain, cerebellum, mesial temporal lobe, and the interface between the cortex and white matter at sulcal depths as well as the corpus callosum (McKee and Daneshvar, 2015, Weiner et al., 2014, Weiner et al., 2017, Tomaiuolo et al., 2004, Maxwell et al., 2006, Hirad et al., 2019, Blecher et al., 2019, Bartsch and Deuschl, 2010, Smith et al., 1997, Ross et al., 1993, Zimmerman et al., 2023, Ghajari et al., 2017) – are also sites of biomechanical vulnerability due to shear strain concentration in healthy participants. We then found that the regions that exhibit high strain, measured with MRE in healthy participants, are the sites of greatest injury, as measured with diffusion MR in acute, sub-acute, and chronic mTBI patients. These findings provide *in vivo* evidence in humans that brain structures differentially vulnerable to high biomechanical strain from skull force loading also exhibit disrupted tissue integrity across the acute, sub-acute, and chronic phases of mTBI recovery. Follow on analyses demonstrate that strain concentration in those regions results from both the differential focusing of impact energy as well as inadequate energy dissipation.

## Highlights

• Normative brain MRE maps identify high-strain “vulnerability” regions.
• High-strain regions overlap with TBI- and CTE vulnerable anatomy.
• Subacute/chronic mTBI shows strain-dependent tissue disruption across the whole brain.
• Acute mTBI shows AFD loss confined to pre-defined high-strain voxels.
• Strain–AFD coupling supports Holbourn’s shear-strain injury hypothesis.

## 1 Introduction

Since Holbourn (1943) it has been hypothesized that shear strain caused by rotational acceleration of the head is the primary mechanical cause of tissue disruption in traumatic brain injury (TBI) (Ommaya et al., 1994, Margulies et al., 1990). While the link between the anatomical distribution of injury and strain, based on Finite Element (FE) modelling has been recently examined in animals (Hajiaghamemar and Margulies, 2021); evidence linking the anatomical distribution of shear strain to patterns of injury from mTBI in humans is lacking. Here we test the prediction: if shear strain is indeed the key factor driving injury in mild TBI (mTBI/Concussion) in humans, then independent measures of the anatomical distribution of shear strain in the human brain will predict the distribution of tissue injury in mTBI.

Research in both animal models and humans has revealed a stereotyped pattern of injury across all severities of TBI, including mTBI, that disproportionately affects midline structures such as the midbrain, cerebellum, mesial temporal lobe, as well as the interface between cortex and white matter at sulcal depths (McKee and Daneshvar, 2015, Weiner et al., 2014, Weiner et al., 2017, Tomaiuolo et al., 2004, Maxwell et al., 2006, Hirad et al., 2019, Blecher et al., 2019, Bartsch and Deuschl, 2010, Smith et al., 1997, Ross et al., 1993, Zimmerman et al., 2023, Ghajari et al., 2017, Gurdjian et al., 1968, Clayton et al., 2012) . Damage to those regions is linked to common mTBI symptoms such as impaired consciousness, balance issues, oculomotor dysfunction, and memory deficits (Zimmerman et al., 2023, Ghajari et al., 2017). While the physiological response to injury involves a complex cascade of neurochemical and electrophysiological dysfunction (Johnson et al., 2013), the initial mechanical disruption caused by shear strain is assumed to be the key driver of anatomically stereotyped patterns of injury (Ommaya et al., 1994, Zimmerman et al., 2023, Ghajari et al., 2017, Hajiaghamemar and Margulies, 2021, Johnson et al., 2013, Arbogast and Margulies, 1998, Corsellis et al., 1973, Cullen et al., 2016, Gennarelli et al., 1982, Graham et al., 1995, Grossman et al., 2012, Omalu et al., 2011, Ommaya, 1995, Palacios et al., 2020). Specifically, it has been argued that the brain areas where injury from mTBI is stereotypically observed are the same areas that are subjected to differentially high concentrations of shear strain^1,15-17,29^.

What is missing is evidence in humans that shear strain is the principal driver of mTBI injury distribution across the whole brain, at least as is measured using widely noninvasive methods such as Diffusion MR (Hirad et al., 2019, Wu et al., 2020, Obenaus et al., 2023, Naess-Schmidt et al., 2018, Lancaster et al., Nov 2018, Hellewell et al., 2021, Chung et al., 2022). To fill this gap, we use Magnetic Resonance Elastography (MRE) to map shear wave propagation and strain concentration in the brain, caused by gentle vibrations applied to the skull with a pneumatic actuator during MRI scanning. We acknowledge there are fundamental differences between low-magnitude, harmonic brain vibrations in MRE studies and the high-magnitude, impulsive head impacts causing mTBI. However, high-impact shear strain depends on specific head kinematics, and is currently not directly measurable, for obvious ethical reasons. In our case we are interested how this noninvasive method captures how strain is distributed across the whole brain *in vivo*. The current investigation is based on the premise that the anatomical pattern of shear strain concentration induced by low-magnitude vibration will be meaningfully related to the anatomical pattern of tissue injury in the setting of high-magnitude head impacts.

We measured tissue disruption in mTBI using Apparent Fiber Density (AFD), a diffusion MRI-based metric that indexes tissue integrity in both white and gray matter (Tournier et al., 2007, Raffelt et al., 2012). Conventional tensor-based measures (FA, MD and related indices) work well white matter regions with coherent fiber directions, but are known to be less reliable in gray matter and in regions with more complex diffusion-restricting compartment architecture (Tournier et al., 2007, Tuch et al., 2002, Afzali et al., 2021). AFD is sensitive to both gray matter and white matter integrity, and is commonly interpreted as reflecting the amplitude (i.e., apparent density) of diffusion-restricting tissue compartments along each resolved orientation (Raffelt et al., 2012, Dhollander et al., 2021, Jeurissen et al., 2014). Relatively lower AFD is therefore taken to indicate loss or disruption of these diffusion-hindering structures (e.g., axons, dendrites, neurites) in that orientation. Animal studies support that premise, showing that decreased AFD corresponds to *histologically confirmed* brain tissue damage in animal studies of TBI (Raffelt et al., 2015, Stampfli et al., 2019). Given that our primary interest is patterns of whole brain strain concentration, CSD modelling and AFD offer a promising approach that provides contrast across both cortical gray matter as well as white matter.

This study uses MRE and diffusion MRI to test three core hypotheses: (1) Holbourns original postulate: the anatomical distribution of shear strain will predict the anatomical distribution of injury in mTBI; (2) shear strain will be highest in regions known to be differentially vulnerable to concussive impacts, such as the midbrain, cerebellum, mesial temporal structures, and sulcal depths; and (3) strain concentration of these regions will be independent of direction of impact (anterior-posterior or lateral-medial).

Secondary hypothesis testing evaluates two nonmutually exclusive mechanisms through which high strain concentration concentrates in specific brain regions. One mechanism is that regions exhibit high shear strain because shear waves concentrate in those regions, perhaps due to the geometry of the skull and voids (ventricles) within the brain parenchyma. A second potential mechanism is that some regions may exhibit high strain because the tissue in those regions is not able to effectively dissipate energy. These two potential mechanisms are not exclusive, and the balance between their contributions to measures of high strain may differ across brain regions.

## 2 Methods

### 2.1 Subacute and chronic mTBI participants

We conducted retrospective analysis of high school and college athletes with a history of concussion and Post Concussive Syndrome (PCS) who had presented between February 2016 and December 2019 to the University of Rochester Medical Center. The University of Rochester Institutional Review Board approved this study, and written informed consent was obtained from all participants. All subjects in our study were referred to the University of Rochester Medical Center outpatient imaging center due to incomplete resolution of symptoms following concussion. All subacute and chronic mTBI patients and controls were scanned on the same 3 T Skyra MRI scanner. Inclusion criteria consisted of a history of concussion and PCS, while exclusion criteria included dental braces, prior brain surgery, ventricular shunt, skull fractures, or other contraindications for MR imaging. Diagnosis of concussion was made by ED physicians initially (recovered from the medical record). PCS was determined through standard clinical consensus across a multi-disciplinary team of neurologists, physical medicine and rehabilitation physicians, and sports medicine physicians. All diagnoses (initial mTBI and subsequent PCS) were made independent of, and blinded to, hypothesis formulation and subsequent inclusion in the analyses of the current report. The control group consisted of age-matched participants with no history of concussion, that were independently collected using the same MRI protocol and scanner, and over the same period.

For retrospective analysis of structural (T1) and dMRI, data from all subjects presenting with concussion from 2016 to 2019 were identified and accessed by a single clinician (SM), and from that point, data were de-identified. Data curation was conducted between June and December of 2022. None of the other study members had access to the identities of the subjects at any point. Number of previous concussions, loss of consciousness at most recent concussion, and time between injury and MRI were determined from review of the electronic medical records. An experienced neuroradiologist (SM) reviewed all MRI examinations, blinded to subsequent hypothesis testing, for any artifacts that might affect study quality, and for the presence of intracranial hemorrhage, signal abnormalities in the brain, hydrocephalus, and congenital or developmental anomalies. That MRI review was performed blinded to all analyses and findings using MRE to identify regions of differentially high versus low strain. 54 participants had dMRI data as well as known time between injury and imaging and could therefore be classified as subacute (delay between injury and imaging, between two weeks and < 90 days, 23 subjects, 14 female, 9 male, age (mean ± SD) = 20.4 ± 2.7) or chronic (delay > 90 days; 31 subjects, 15 female, 16 male, age (mean ± SD) = 20.7 ± 3.8).

### 2.2 Acute mTBI cohort

We conducted retrospective analyses on 15 male and 14 female mTBI patients (mean age = 19.5, median age = 19) and 15 male controls (mean age = 21.6, median age = 21). The individuals diagnosed with concussion represented a subset of a broader group of NCAA contact-sport athletes at the University of Rochester and the Rochester Institute of Technology who were monitored for concussion. Between 2009 and 2014, National Collegiate Athletic Association (NCAA) division I and III collegiate contact sport athletes were followed prospectively for a diagnosis of mTBI (Hirad et al., 2019, Bazarian et al., 2012, Bazarian et al., 2014). mTBI was defined as an injury witnessed by an on-field certified athletic trainer and meeting the definition of concussion as defined by the Sport Concussion Assessment Tool 2. The diffusion MRI scans were collected within 72 h post-injury on concussed individuals (Hirad et al., 2019). As such, diagnosis of mTBI was made independent of decisions about including those participants in the current study. The University of Rochester Institutional Review Board approved this study, *as an exempt study for secondary use of pre-existing data*.

### 2.3 MRI acquisition for subacute and chronic mTBI cohorts and matched controls

MR imaging was conducted on a Siemens Skyra 3 T scanner using a 20-channel head and neck coil. Diffusion imaging was performed with a b-value of 1000 s/mm^2^, using 64 diffusion-encoding directions. In addition, a b = 0 s/mm^2^ image was collected. Additional dMRI parameters included: FOV = 256 x 256 mm, 70 slices, image resolution = 2x2x2 mm^3^, TR/TE = 9000/88 ms, GRAPPA factor = 2, acquisition time = 10 min and 14 s. A GRE sequence was also collected with TEs = 4.92, 7.38 ms at the same resolution of the dMRI to correct for susceptibility-induced distortion. The MRI protocol also consisted of T1 magnetization-prepared rapid gradient echo (MP-RAGE) (TR = 1200 ms, TE = 2.29 ms, TI = 600 ms, flip angle = 8 degrees, FOV = 250 mm, 1 x 1 x 1 mm^3^, 208 slices), 3D Axial SWI (TR = 27 ms, TE = 29 ms, FOV = 220 mm, 1.5 mm slice thickness, 88 slices), and Double IR fat suppressed FLAIR (TR = 7500 ms, TE = 321 ms, TIs = 3000, 450 ms, FOV = 260 mm,1.4 mm slice thickness, 120 slices), which were not used in the current study, but were collected as part of standard clinical care.

### 2.4 General procedures and MRI acquisition parameters for the acute mTBI cohort and age, and scanner-matched controls

Participants were tested on a Siemens 3 T Trio scanner using a 32-channel head coil at the Rochester Center for Brain Imaging (now named: Center for Advanced Brain Imaging and Neurophysiology). High-resolution structural T1 contrast images were acquired using a magnetization-prepared rapid gradient echo (MP-RAGE) pulse sequence at the start of each participant’s first scanning session (TR = 2530 ms, TE = 3.44 ms, flip angle = 7 degrees, FOV = 256 mm, matrix = 256 x 256, 1x1x1 mm sagittal left-to-right slices). DTI sequence parameters were: TR/TE = 10 s/89 ms, voxel size 2x2x2 mm, 60 diffusion directions with b = 1000 s/mm2 and 10 averages of b = 0.

### 2.5 Diffusion MR analysis

Fieldmaps were generated from the GRE data using the fugue command in FSL (Winkler et al., 2014). The fieldmaps were unwrapped and used to correct susceptibility-induced distortions. The FSL eddy command was used to correct for effects of eddy currents as well as subject motion (Andersson and Sotiropoulos, 2016). The data was subsequently bias-field corrected using ANTs (http://stnava.github.io/ANTs/).

The *DIPY* toolbox (version 1.1.1, https://www.dipy.org) was used to calculate DTI and fODF metrics. We first computed conventional DTI metrics and then estimated the fiber orientation distribution (fODF) using constrained spherical deconvolution (CSD) on the single-shell b = 1000 s/mm^2^ acquisition. CSD captures within voxel angular structure (including multiple orientations) that is not modeled by a single diffusion tensor (Tournier et al., 2007, Raffelt et al., 2012, Jeurissen et al., 2014). A single-tissue, single-shell CSD model was fit using a white matter response function calibrated from high-FA voxels in each subject. Within each voxel, the fODF provides an orientation-resolved representation of the diffusion-weighted signal as a function of direction. We then derived AFDtotal as an amplitude-based, voxel-wise metric defined as the zeroth-order (*l*=0) spherical-harmonic (SH) coefficient of the fODF (i.e., the spherical-mean / “mass” term) (Li et al., 2019). The isotropic (*l* =0) term of the fODF tends to track the overall amount of diffusion-hindering tissue in a voxel (Raffelt et al., 2012). At sufficiently high b-values, AFDtotal has been shown to approximate intra-axonal water fraction (Dhollander et al., 2021, Jeurissen et al., 2014, Li et al., 2019), however at *b*= 1000 s/mm^2^_,_ the b value of our study, we interpret AFDtotal conservatively as an orientation-independent, amplitude-based measure (i.e., an apparent density metric) of diffusion-hindering, extracellular signal, rather than a specific intra-axonal volume fraction. As such, in this study, we make no claims regarding intra-axonal volume fraction related changes following mTBI. However, the purpose of this study is to determine whether the anatomical patterns of MRE-based whole brain strain distribution predict whole brain patterns of diffusion MR measured tissue injury. The combination of b value and AFDtotal provides a robust, whole brain scalar contrast with sufficient gray and white matter signal-to-noise for voxelwise and ROI-level group comparisons, while avoiding the additional assumptions and data requirements needed for compartment-specific (multi-shell / multi-tissue) modeling. In particular, b = 1000 s/mm^2^ preserves substantial tissue signal in both cortex and deep white matter (while CSF is already strongly attenuated) (Jeurissen et al., 2014). Furthermore, AFDtotal (the l = 0 “mass” term of the fODF) offers an orientation-independent summary (Li et al., 2019) that is well suited to our aim of cross-tissue, strain-stratified injury mapping, rather than tractography or fixel-resolved intra-axonal quantification. Accordingly, we interpret group differences in AFDtotal as reflecting changes in an amplitude-based diffusion-hindering, tissue-like signal contrast across the brain, consistent with our primary objective of testing whether MRE-derived strain distributions predicts the anatomic pattern of diffusion MR based measures of tissue alteration after mTBI. Throughout this manuscript, “AFD” refers to the *l*=0 fODF term (AFDtotal).

For group-level analyses, native space diffusion images were nonlinearly registered to MNI152 1 mm3 space. Briefly, each participant’s FA map was nonlinearly registered to the FMRIB58_FA template in MNI space using the TBSS nonlinear registration framework, and the resulting warp fields were applied to the AFD maps using tbss_non_FA function (www.fmrib.ox.ac.uk/fsl/data/FMRIB58_FA) (Hirad et al., 2019). All diffusion MRI analyses (including high-strain vs low-strain ROI comparisons) and all MRE analyses (see below) were conducted in this common MNI152 1 mm^3^ space.

### 2.6 MRE data acquisition

MRE data were obtained from publicly available depositions collected in two separate cohorts of healthy control participants. The first cohort consisted of 59 subjects (33 females, and 26 males, Average Age of 37.4, Range 15–75) and the data were collected at, and made publicly available by, the University of Delaware (UDEL) using a Siemens 3 T Prisma scanner with a 64-channel head/neck coil (complete protocol available at ref^49^). Skull displacements were generated using a resoundant acoustic (Resoundant Acoustic Driver System, Resoundant™ Rochester, MN) driver system with a soft pillow occipital actuator at 50 Hz. A 3D multiband, multishot spiral sequence was used to measure brain tissue displacements, providing whole-brain coverage with an imaging resolution of 1.5 mm isotropic (240 x 240 x 120 mm^3^).

The second MRE cohort consisted of 24 subjects (12 females, and 12 males, Average Age of 37.2, Range 20–68), and the MRE data were obtained from the same public repository but collected at the University of Washington St. Louis (WUSTL) (Bayly et al., 2021). Participants underwent harmonic lateral skull actuation at 50 Hz using the same Resoundant actuator system using a passive flexible silicone bottle at the lateral skull. The lateral actuation approach provided a different pattern of skull motion compared to the occipital actuation used in the UDEL dataset. MRE data for the WUSTL cohort was acquired at a lower isotropic voxel resolution of 3 mm, compared to the 1.5 mm isotropic resolution of the UDEL cohort.

Both material properties and strain data were provided in NIFTI format in the database for UDEL and only the strain data was provided for the WUST site. Briefly, the team providing the data used an NLI inversion algorithm to estimate complex shear, storage, and loss moduli (complex viscoelastic shear modulus (G* = G’+iG”), with G’ as the storage modulus and G” as the loss modulus) (McGarry et al., 2012). Shear stiffness and damping ratios were calculated from those properties (see ref^49^). Additionally, octahedral shear strain (OSS) was derived at each voxel based on the strain tensors of that voxel, and represents the maximum shear strain regardless of direction at the location (McGarry et al., 2011) (see ref 38 for details). Finally, in our analysis we used OSS as the main variable for shear strain, damping ratios (ξ = G”/2G) as measure of energy dissipation and storage modulus as a measure of energy absorbance.

### 2.7 MRE processing and analysis

The analysis was conducted in MNI152 1 mm^3^, (Montreal Neurological Institute) space, same space as the diffusion MRI data, to facilitate the generation of group-level strain concentration maps and support extensionality for testing in the separate cohorts of mTBI participants. For the MRE data, we follow the standard-space pipeline described by Hiscox and colleagues, which underlies the current reference atlas for brain viscoelastic properties (Hiscox et al., 2020). Briefly, each participant’s T1-weighted MPRAGE and MRE magnitude images undergo brain extraction (FSL BET). The T1 is then nonlinearly registered to MNI152 1 mm^3^ space using ANTs SyN tool (Advanced Normalization Tools, http://stnava.github.io/ANTs/). The MRE magnitude image is first rigidly/aligned to the subject’s T1, and the resulting transforms (rigid/affine and nonlinear warp) are concatenated and applied to the MRE-derived mechanical maps, bringing all MRE data into MNI152 1 mm3 space. This transformation allowed for the analysis of strain concentration at the group level, enabling comparisons and statistical evaluation across participants and cohorts.

### 2.8 Registration quality assurance

Registration quality was assessed for every subject by visual overlay of (i) MRE magnitude and T1 images in native space and (ii) MRE-derived strain maps and AFD maps on the MNI152 template, with particular attention to gray–white matter boundaries and major sulci and gyri. Datasets with clear misregistration (e.g., visible mismatch of the cortical ribbon or ventricles) were to be excluded from further analysis; however, no subjects met these exclusion criteria.

### 2.9 Statistical analysis of strain distribution in relation to diffusion metrics

**Fig. 1:**
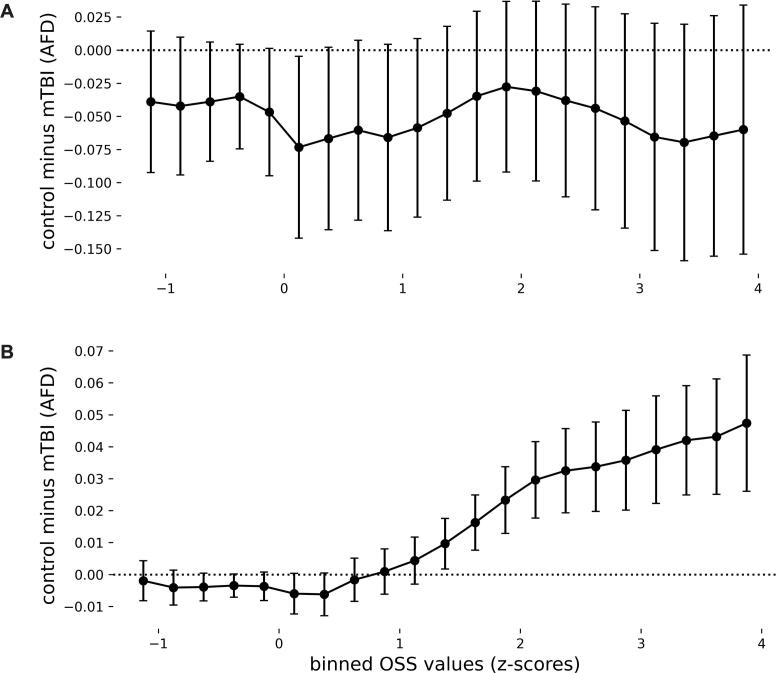
Threshold-agnostic whole-brain analyses demonstrate a positive relation between strain concentration and tissue integrity in the subacute and chronic, but not acute, phases of mTBI. *A)* Acute cohort. For each subject, apparent fiber density (AFD) was averaged within anatomical bins of normative octahedral shear strain (OSS) defined from the independent MRE cohort (0.25-width OSS z-score bins in MNI152 space). The curve shows the mean difference in AFD between controls and acute mTBI (control – mTBI) as a function of OSS bin; error bars indicate the 95% confidence interval across subjects. Values near zero across the OSS range indicate no detectable strain-dependent group difference in AFD at the whole-brain level within 72 h of injury. *B)* Subacute/chronic cohort. Same analysis for the combined subacute and chronic PCS mTBI cohort and their controls. Here, control – mTBI AFD differences are near zero in low-OSS bins and increase monotonically at higher OSS, indicating that regions that normatively experience greater shear strain show larger reductions in AFD in mTBI compared with controls. Formal mixed-effects statistics for these patterns are reported in the main text.

To quantify strain-related tissue disruption, we derived a measure of “differential AFD” (ΔAFD) as our dependent variable, by subtracting mean AFD values between cohorts and regions (Fig. 1. control – mTBI; Fig. 3. high-strain – low-strain). This ΔAFD approach is directly analogous to the ΔFA, %ΔFA, and voxel-wise difference methods widely used in concussion and subconcussion DTI studies, where pre–post or between-group differences in FA are computed and related to head-impact exposure or strain metrics (Bazarian et al., 2012, Bazarian et al., 2014, Bahrami et al., 2016, Davenport et al., 2014, Holcomb et al., 2021), including in our own prior work on midbrain diffusion changes in concussion and subconcussion (Hirad et al., 2019). We then followed two complementary approaches to model shear strain as the independent variable.

**Fig. 2:**
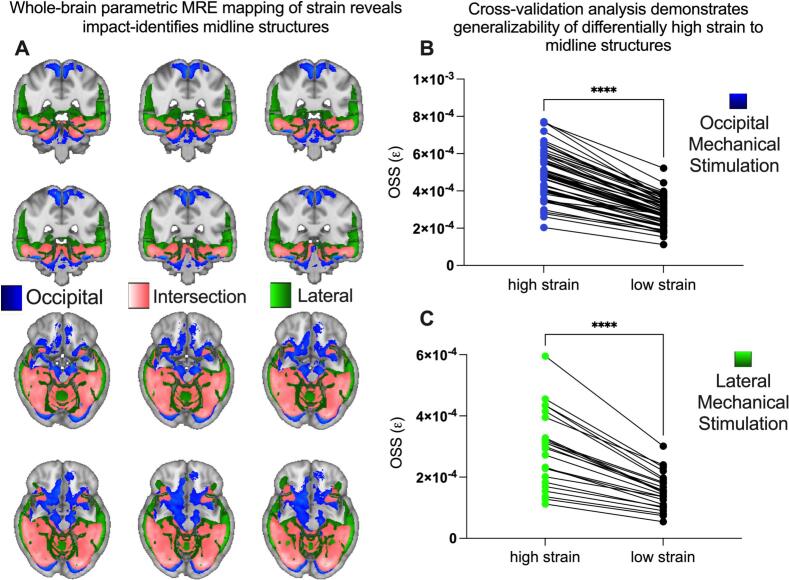
Differentially high strain concentration in specific brain structures caused by 50 Hz occipital and lateral actuation of the head, measured using MRE. *A)* Strain concentration from occipital head actuation (blue), lateral head actuation (green), and regions of intersection (pink) between occipital and lateral actuation. Pink regions experience differentially high strain concentration that is invariant to the location of skull actuation. *B)* Leave-one-out cross-validation analysis of the occipital actuation MRE data (n = 59) demonstrating differentially increased strain concentration across *all* subjects. *C)* Leave-one-out cross-validation analysis of the lateral actuation MRE data (n = 24) demonstrating strain concentration across *all* subjects. (For interpretation of the references to colour in this figure legend, the reader is referred to the web version of this article.)

**Fig. 3:**
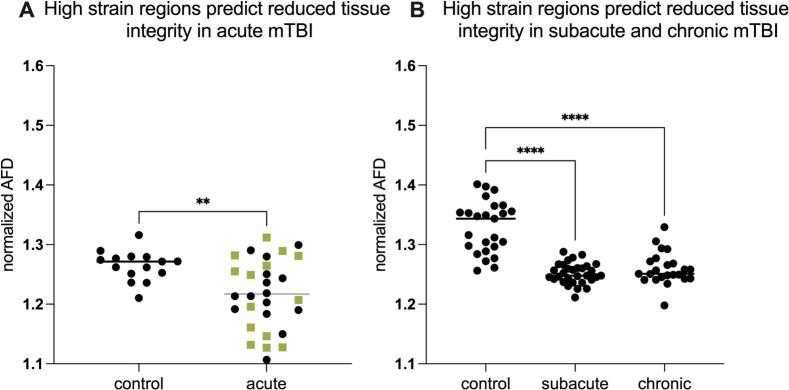
High strain concentration, measured in healthy participants using MRE, predicts regions of reduced tissue integrity in mTBI patients. Y axes represent AFD values in high strain regions normalized to low strain regions (within participants). As described in the text, 2 (mTBI vs Controls) x 2 (High- vs Low-Strain Regions) ANOVAs demonstrated significant interactions in each mTBI sub-group. *A)* There was significantly lower Apparent Fiber Density (AFD) in high-strain regions normalized to low-strain regions (AFD_HS_/AFD_LS_) in acute mTBI compared to control subjects (olive green squares are female mTBI patients, separated because the controls are entirely male). *B)* There was differentially lower AFD in high-strain regions normalized to low-strain regions (AFD_HS_/AFD_LS_) in subacute and chronic mTBI participants, compared to controls. (For interpretation of the references to colour in this figure legend, the reader is referred to the web version of this article.)

### 2.10 Subject-level OSS binning (threshold-agnostic)

MRE-derived OSS is available in an independent healthy cohort, and was used to compute a normative OSS z-score map. This was created by averaging OSS across healthy subjects at each voxel. We then discretized this normative OSS z-score map into bins of width 0.25 z-score units spanning the full observed range (approximately ranging from − 1 to + 4), assigning each voxel to a bin based on its normative OSS z-score. This map serves as a fixed spatial regressor that defines the population-level “mechanical vulnerability” of each voxel. For the diffusion data, each subject’s AFD map (already registered to MNI152 1 mm^3^ space) was sampled within these OSS bins. For every subject (mTBI or control) we computed the mean AFD within each OSS bin, so that the unit of analysis is the subject, not the voxel. This yields, for each cohort, a set of subject-level AFD values across OSS bins. We then summarized, for each bin, the mean and 95% confidence interval of AFD in controls and mTBI cohorts, and expressed “differential reduction” as the difference in mean AFD between groups (control – mTBI) as a function of the OSS bin (Fig. 1). This approach reduces the impact of voxel-to-voxel variability in AFD and directly tests whether regions that are normatively exposed to higher shear strain show greater AFD loss in mTBI than in controls.

To formally test the strain–AFD relationship at the subject level, we fit a linear mixed-effects model separately in the acute and subacute/chronic cohorts of the form,

AFD_mean_ ∼ group x OSS_bin_center + (1|subject),

with random intercepts for subjects and OSS_bin_center treated as a continuous predictor. From this model we report slopes, 95% confidence intervals, and p values for the group, OSS, and group × OSS interaction terms, providing subject-level inference on whether AFD systematically varies with shear strain, and differentially in mTBI versus controls.

### 2.11 High- vs low-strain regions of interest (HS vs LS)

To identify high-strain voxels, we computed a voxelwise one-sample *t*-test across MRE subjects on the OSS z-score maps (H_0_: mean OSS z-score = 0) within the MNI brain mask. The resulting p-values were corrected for multiple comparisons using Benjamini–Hochberg FDR, and voxels with FDR-adjusted p < 0.05 (q < 0.05) were initially considered significant. We imposed an additional effect-size criterion and defined the final high-strain ROI as the set of voxels satisfying both q < 0.05 and t ≥ 4. All downstream ‘high-strain’ analyses were restricted to this joint FDR- and t-thresholded mask. The low-strain (LS) region was defined as the complement of HS within the brain mask, i.e., all voxels that did not satisfy the joint q < 0.05 and t ≥ 4 criteria (including voxels with OSS z-scores near zero). Thus LS represents the rest of the gray- and white-matter volume and serves as a non–high-strain comparison region.

## 3 Results

### 3.1 Shear strain predicts reductions in structural integrity

We first examined how normative strain concentration relates to regional AFD in a whole-brain OSS-binning analysis, performed separately for the acute and subacute/chronic cohorts (Fig. 1). In the acute cohort, control–mTBI difference remained close to zero across bins (Fig. 1A) and both groups showed a similar increase of AFD with OSS (Supplemental Fig. 1A). As such, the subject-level binning and mixed-effects model show no significant group main effect or group × OSS interaction (mTBI slope ≈ 0.043, 95% CI 0.040–0.046; control slope ≈ 0.041, 95% CI 0.037–0.044; group effect at mean OSS β≈ −0.051, 95% CI − 0.137–0.036, p = 0.25; interaction β≈ −0.002, 95% CI − 0.007–0.002, p = 0.29). At this early time point, the OSS–AFD scaling is therefore similar in mTBI and controls at the whole-brain level, indicating that strain-dependent group differences, if present, are not detectable within the first 72 h with this diffusion model.

In contrast, in the subacute/chronic cohort, our analysis showed that group differences in AFD are near zero in low-OSS bins and become progressively larger at higher OSS, indicating that regions with greater normative strain concentration exhibit greater reduction in tissue integrity in mTBI compared to controls (Fig. 1B). AFD increased with OSS in both groups but more steeply in controls (Supplemental Fig. 1B), yielding a significant group × OSS interaction (mTBI slope ≈ 0.029 per OSS z-score, 95% CI 0.028–0.031; control slope ≈ 0.041, 95% CI 0.039–0.044; interaction β≈ 0.012, 95% CI 0.009–0.015, p < 0.001). This supports the core premise of Holbourn’s 1943 postulate that *“shear-strain present at any point in the brain should be…a rough measure of the probability of injury at that point”.*

### 3.2 Testing if brain regions exhibiting high strain concentration are those known to be stereotypically involved in mTBI injury

MRE data were analyzed from 59 healthy control subjects who underwent occipital mechanical stimulation at 50 Hz, and 24 subjects who underwent lateral mechanical stimulation at 50 Hz (Bayly et al., 2021). Our core analyses were focused on 50 Hz stimulation, because it is the most commonly used frequency for MRE data collection, and it corresponds to the largest possible sample size involving both occipital and lateral stimulation (all findings were qualitatively replicated at 30 and 70 Hz; see Supplemental Fig. 2). For each subject, OSS was converted to z-scores, normalized within subject, across all voxels, and analyzed in MNI152 1 mm^3^ space. Normatively defined high-strain (HS) and low-strain (LS) voxels, based on healthy participants, were defined separately, as described in the Methods.

For both lateral and occipital mechanical stimulation, the midbrain, cerebellum, and mesial temporal lobe (hippocampus and parahippocampus) exhibited differentially high strain concentration (Fig. 2A–B). In agreement with prior histopathologic findings in chronic traumatic encephalopathy, the depths of the cortical sulci also showed pronounced strain concentration (Ghajari et al., 2017, McKee et al., 2013). Regions like the genu and splenium of the corpus callosum, known to be vulnerable to TBI related strain and injuries (Tomaiuolo et al., 2004, Post et al., 2017, McAllister et al., 2012, Champagne et al., 2019, Adams et al., 1989, Sharp et al., 2011), were also identified as regions exhibiting differentially high strain concentration (see Supplemental Table 1 for all regions with ≥ 10 voxels). Quantitatively, the HS mask occupied 10.7% of the total brain volume and contained 15.8% of all gray-matter voxels and 13.0% of all white-matter voxels in the MNI152 brain. Within the HS mask itself, 59.2% of voxels were gray matter and 40.8% were white matter, indicating that high-strain regions involve both cortical and subcortical tissue rather than being dominated by a single tissue class. These findings confirm the hypothesis that regions exhibiting high strain concentration based a normative MRE map correspond closely to structures that are stereotypically involved in mTBI-related injury.

### 3.3 Cross-validation analysis

In order to quantify the generalizability of the Strain Concentration Analysis, we conducted a leave-one-out cross-validation of the MRE data. High Strain Regions were defined at t = 4.0 (FDR-corrected q < 0.05) on group data from *n-1* participants, and the resulting ROIs for high and low-strain regions were used to extract OSS from the left-out participant (folded over all participants). The results indicate a robust and consistent pattern of differentially high strain concentration in the same set of regions, across all subjects, for both lateral (t = 10.3, p < 0.0001) and occipital actuation (t = 23.5, p < 0.0001; Wilcoxon matched-pairs rank test, Fig. 2 B and C). These data support the existence of stereotyped patterns of high strain concentration in specific brain structures independent of whether the mechanical stimulation of the skull was lateral or occipital.

### 3.4 The anatomical distribution of high strain concentration predicts the distribution of tissue injury in acute, subacute, and chronic mTBI

We then test whether regions that exhibit differentially high strain concentration also exhibit differential injury in mTBI, across a wide timeline of recovery: within 72 h of injury (acute, n = 29), between 2 weeks and 90 days after injury (subacute, n = 31), and more than 90 days post-injury (chronic, n = 23). For each mTBI cohort, dMRI data from the same scanner, and scanning parameters, were collected from an age-matched control group without a history of TBI. We extracted Apparent Fiber Density (AFD) at each voxel in each subject (mTBI participants and matched controls). AFD values were averaged for regions identified in the MRE analyses as exhibiting high strain and separately for regions exhibiting low strain. High Strain Concentration regions were defined based on MRE data as the intersection of high-strain regions observed *both for* posterior (occipital) and lateral skull perturbations.

This analysis has high rigor and reproducibility because ROIs are defined in a group of 83 healthy controls using stringent criteria and then tested (in stereotactic space) in three independent TBI cohorts, covering the full timeline of post-concussive recovery (acute, subacute, and chronic). Three planned 2_[high vs. low strain]_ X 2_[mTBI vs control]_ ANOVAs, with AFD as the dependent measure, were conducted for the acute, sub-acute, and chronic mTBI patients. Critically, the interaction terms were significant for all three post-concussive groups: acute (F = 4.63; P < 0.037), subacute (F = 60.79; P < 0.0001), and chronic (F = 31.38; P < 0.0001). Thus, the differential findings between patients and controls are predominantly driven by these high-strain regions, supporting their significance in mTBI injury patterns. To test the directed hypothesis that high strain regions exhibit lower AFD compared to low strain regions, we first computed the ratio AFD(High)/AFD(Low) for each participant, a normalized measure that accounts for baseline variability and focuses on relative differences between high and low strain ROIs. That directed hypothesis test was significant for each of the mTBI groups vs. their respective controls: acute (t = 3.0; p < 0.005) Fig. 3A; subacute (t = 9.0, p < 0.0001) and chronic (t = 6.6; p < 0.0001) Fig. 3B). In the acute cohort, all controls (n = 5) were male whereas the mTBI group included both males (n = 15) and females (n = 14). To assess whether this sex imbalance could account for the effect, we repeated the analysis stratified by sex; the high-strain AFD reduction persisted in both acute males (control vs. acute male: t = 3.13, p < 0.005) and acute females (control vs. acute female: t = 2.59, p < 0.015). Together, these tests demonstrate that high strain regions show significantly lower AFD in mTBI groups compared to controls, while low strain regions exhibit no significant difference in AFD between mTBI and controls; these finds align with the hypothesis that strain concentration in drives tissue vulnerability in regions stereotypically involved in injury from mTBI.

### 3.5 Both differential energy and a differential reduction in dissipation of energy drive high strain concentration

**Fig. 4:**
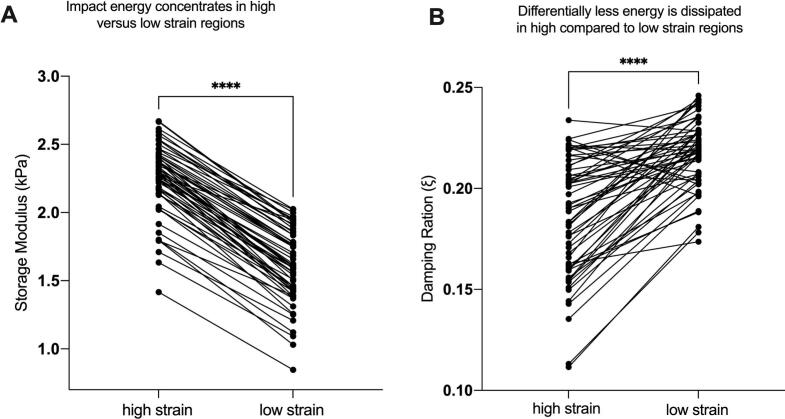
Differentially high strain is caused both by differential focusing of impact energy and reduced dissipation of that energy. *A)* Given the high strain regions have also higher storage modulus, indicates that hear wave energy is differentially focused in regions of high strain concentration. *B)* The regions with impact-location invariant high strain concentration have lower damping ratio compared to low strain regions. These findings mean that differentially high strain is due to *both* higher energy loading of those brain structures and the inability of those brain structures to dissipate that energy.

As discussed in the introduction, high strain concentration in specific brain structures could be caused by two non-exclusive reasons: those structures experience high levels of shear wave forces (higher energy), and/or those regions are differentially stiff compared to surrounding brain structures, rendering them unable to dissipate the energy that arrives. We sought evidence for each of these alternatives in parallel, as they are non-exclusive biomechanical accounts of the proximate cause of differential injury observed in high strain regions. Storage modulus was used as the main measure of energy absorbance (n = 59). We found that there is greater storage modulus in high strain regions compared to the rest of the brain (t = 38.71, p < 0.0001; Fig. 4A). *These findings reveal that, despite being stiffer than the rest of the brain, these regions experienced greater strain due to a higher concentration of forces*.

We also tested whether regions exhibiting differentially high strain (and higher loading) also exhibit a reduced ability to dissipate the shear energy that arrives, compared to low-strain brain regions. To do that, we compared the damping ratios of the high strain regions to that of the low strain. We found that high-strain brain regions exhibited lower damping ratios, compared to low strain regions (t = 9.24, p < 0.0001; Fig. 4B). *This indicates that the viscoelastic properties of brain regions that exhibit injury in mTBI are also differentially unable to dissipate energy from external skull loading*.

## 4 Discussion

Holbourn (1943) proposed that the brain is particularly vulnerable to shear strains because of its low modulus of rigidity and postulated that “shear-strain present at any point in the brain should be….a rough measure of the probability of injury at that point” (Holbourn, 1943). For the intervening 80 years, the field has largely worked within the framework of Holbourn’s postulate, in arguing that the principal proximate cause of injury in TBI is due to strain forces damaging brain tissue. Here, we have applied mechanics-based concepts to quantitatively test whether the patterns of shear force concentration in the brain predicts the anatomical distribution of tissue disruption in mTBI. First, we showed that in the subacute/chronic cohort, brain regions that normatively experience higher shear strain on MRE exhibit disproportionately greater reductions in AFD, indicating greater loss of tissue integrity in mTBI. This finding supports Holbourn’s postulate that shear strain concentration predicts the distribution of injury (Fig. 1B). In contrast, in the acute cohort the relation of OSS to AFD was similar in mTBI and controls at the whole-brain level (Supplemental Fig. 1A), and strain-dependent group differences between groups were not detectable within the first 72 h with this diffusion model (Fig. 1A). Next, we used MRE to show that the midbrain, cerebellum, mesial temporal lobe structures, sulcal depths, and genu and splenium of the corpus collosum exhibit differentially high strain concentration (Fig. 2A). Those regions are known for exhibiting differentially high levels of tissue injury in mTBI, and disruption to the normal functions of those regions has been related to the common signs and symptoms of mTBI (Zimmerman et al., 2023, Ghajari et al., 2017). To empirically validate the biomechanical measurements obtained in healthy participants using MRE, we tested if regions exhibiting high strain concentration are also those that exhibit reductions in tissue integrity in acute, sub-acute and chronic cohorts of mTBI participants (compared to respective control groups; Fig. 2A and B). We observed a consistent pattern of differential injury in high-strain regions compared to low-strain regions, in acute, sub-acute, and chronic subgroups of mTBI patients. Finally, we sought positive evidence for each of two nonexclusive biomechanical causes of high strain concentration in specific brain structures: shear waves from skull impacts differentially concentrate in certain regions, and/or, certain regions are less able to dissipate arriving shear wave energy. We found evidence that there is indeed differential focusing of shear forces leading to higher energy transmission to high-strain regions (Fig. 4A), and that those regions also dissipate less energy relative to low strain regions (Fig. 4B).

Our results suggest a temporal evolution in how shear strain relates to diffusion-defined tissue changes. In the subacute/chronic PCS cohort, higher normative shear strain is associated with progressively greater AFD reductions across the full OSS range, yielding a clear strain-dependent group difference at the whole-brain level (Fig. 1B). By contrast, in the acute cohort the relation of OSS and AFD is similar in mTBI and controls when examined across all voxels (Fig. 1A and Supplemental Fig. 1A), yet AFD reductions are detectable when the analysis is restricted to the pre-defined high-strain mask (Fig. 3A). This pattern is consistent with the idea that, acutely, strain-related microstructural changes are relatively small and spatially confined to the most mechanically vulnerable voxels (t ≥ 4, comprising ∼ 10.7% of brain volume). For that reason, by hypothesis, such changes are detectable in a targeted high-strain ROI but do not yet manifest as a robust gradient across the entire spectrum of strain. By the subacute/chronic stage, the injury pattern appears to broaden, and strain-dependent group differences emerge across OSS bins. This temporal evolution is in line with longitudinal diffusion MRI studies showing that white-matter abnormalities after mTBI can be subtle or spatially restricted early after injury, and become more pronounced over subacute and chronic time scales (Palacios et al., 2020, Yin et al., 2019). It also parallels finite element–based work in collision-sport athletes, where cumulative regional strain metrics over a season predict diffusion changes even in the absence of diagnosed concussion (Holcomb et al., 2021, Miller et al., 2022). Given the modest sample sizes, especially in the acute cohort, we caution against strong conclusions about whether the strain–AFD relationship is intrinsically weaker in mTBI cases that do not go on to develop persistent symptoms. As such, larger, longitudinal studies that follow both PCS and non-PCS trajectories will be needed to resolve this question. Moreover, future studies with diffusion MRI acquisitions optimized for multi-shell / multi-model analyses should explore how DKI, NODDI, or related metrics compare to, or complement, the AFD effects we report here.

Prior work using computational modelling has demonstrated the anatomical specificity of strain concentration and its relationship to injury patterns in TBI. Ghajari and colleagues (2017) used computational modeling to show that the greatest strain and strain rate are concentrated in sulcal depths, regions that frequently exhibit neuropathology in chronic traumatic encephalopathy (CTE) (Ghajari et al., 2017). Their findings, derived from simulations of various biomechanical loading conditions, align closely with our observations of high-strain concentration in the sulcal depths, midbrain, and corpus callosum. Moreover, seminal animal work by Hajiaghamemar and colleagues (2021) demonstrated that finite element model-derived strain metrics accurately predict diffuse axonal injury locations, underscoring the utility of strain-based measures for localizing injury (Hajiaghamemar and Margulies, 2021). In human cohorts, finite-element–derived strain metrics have also been linked directly to diffusion MRI changes. McAllister and colleagues showed that maximum principal strain and strain rate correlate with DTI measures of corpus callosum integrity in concussed football players (McAllister et al., 2012); Holcomb and colleagues related cumulative tensile strain and strain rate to regional diffusion changes in youth football players (Holcomb et al., 2021); and Miller and colleagues demonstrated that cumulative strain-based metrics predict exposure-related imaging changes, including diffusion measures, in youth football players (Miller et al., 2022). Our work builds upon these modelling studies in humans and animals by empirically demonstrating that regions with high strain concentration, as identified through MRE, are the same regions exhibiting reduced tissue integrity, as measured with AFD across acute, sub-acute, and chronic phases of mTBI.

An important difference between the current investigation and prior studies that have used finite element modeling (FEM) is our use of Octahedral Shear Strain (OSS) instead of Maximum Principal Strain (MPS) as a metric in assessing injury tolerance and distribution (Zhan et al., 2024). MPS, derived as the first eigenvalue of the Green-Lagrange strain tensor, encompasses both shear and longitudinal strain components. While MPS has been extensively employed in injury prediction, its composite nature does not isolate shear strain specifically. In this study, we adopted OSS to focus on shear strain dynamics, to explicitly test the relation of shear strain and tissue injury. Nonetheless, it is important to underline that OSS and MPS were highly correlated at the voxel level across all 59 subjects in our dataset (R^2^ of 0.83).

Previous studies have highlighted the biomechanical vulnerability of midline structures to injury in the setting of TBI. The brainstem, for example, is believed to be susceptible to TBI due to its location and geometry relative to the cortex, resulting in differential sensitivity to rotational loading (Arbogast and Margulies, 1998). FEM of video recordings of concussive episodes in the NFL have inferred rates of high strain in the midbrain, thalamus, and hippocampal/parahippocampal gyrus, accounting for concussions observed in American football players (Viano et al., 2005). A comprehensive review of 1280 National Football League games identified for detailed video analysis: 20 head impacts displaying evident signs of loss of consciousness, 21 with abnormal posturing, and 41 control impacts devoid of observable neurological signs. Those videos were used to guide physical reconstructions of the impacts to estimate impact kinematics (Zimmerman et al., 2023). That detailed analysis revealed that impacts associated with loss of consciousness displayed significantly higher head acceleration and brain deformation in various regions, notably the midbrain and cerebellar areas, suggesting biomechanical vulnerability of brainstem nuclei that are instrumental in maintaining consciousness. In line with this, previous research from our group has shown that the amount of rotational loading experienced by collegiate American football players, as measured by helmet-worn accelerometers, predicts the extent of axonal injury in the midbrain, even in the absence of concussion (Hirad et al., 2019). These observations also align with prior Finite Element modeling studies that found strain concentration in deep brain structures is due to modal dynamics and nonlinear material properties (Clayton et al., 2012, Laksari et al., 2020). Furthermore, the overlap of high-strain regions across 30, 50, and 70 Hz (Supplemental Fig. 2) underscores the robustness of these findings and highlights regions such as sulcal depths and the corpus callosum as consistently vulnerable to strain across a range of frequencies. This frequency-invariant overlap may be particularly significant given the established role of these regions in mTBI pathology, as evidenced by post-mortem histopathological studies and *in vivo* imaging data from CTE cases.

MRE allows for the quantitative measure of material and geometric discontinuities that cause shear strain in soft tissues such as the brain (Muthupillai et al., 1995, Kruse et al., 2008, Sack et al., 2008, Green et al., 2008). In line with established principles of mechanics, MRE-based measures in soft tissue indicate that strain concentrates at interfaces between materials of different viscoelastic properties (Okamoto et al., 2019, Yin et al., Nov 2015). Our data align with findings showing strain concentration in the depths of sulci and the propagation of shear waves through midline structures in a healthy control subject (McCracken et al., 2003, McCracken, 2005). Although not explicitly stated by the authors, tagged-MRI data from the Bayly group at WUSTL also appears to align with our findings of strain concentration in the midbrain and within sulcal depths (Alshareef et al., 2021, Sabet et al., 2008, Bayly et al., 2005). Our work further aligns with modelling studies that show sulcal depths tend to experience relative high strain (Ghajari et al., 2017). Furthermore, Escarcega and colleagues (2023) demonstrated that strain fields derived from low-frequency MRE harmonics closely resemble those observed in tagged MRI during impulsive loading, underscoring the potential of MRE to illuminate deformation patterns associated with impulsive events (Escarcega et al., 2023).

Mechanical theories predict that failure of a structure occurs when the deformation of that structure exceeds a threshold beyond which the material integrity of the structure is undermined. In biological organs, such as bones, such concentrations arise from stiffness discontinuities between heterogeneous structures (Burr, 2019, Hernandez and van der Meulen, 2017, Kasiri and Taylor, 2008). Our findings suggest that the brain, with its heterogeneous geometry, numerous interfaces, and viscoelastic discontinuities, has naturally occurring regions of differentially high strain concentration. To our knowledge, this study represents the first comprehensive demonstration that strain concentration maps, indexing biomechanical vulnerability independent of impact location, and obtained with *in vivo* measurements in humans, can identify regions that exhibit stereotyped injury in mTBI.

The existence of stereotyped regions of high strain concentration in the human brain has implications for the interpretation of prior histopathology studies of brain injury caused by subclinical repetitive traumatic impacts, such as those sustained in the setting of contact sports and military operations (Omalu et al., 2011, McKee et al., 2013, Omalu et al., 2006, Omalu et al., Nov 2011). Brain regions representing differentially high levels of strain are likely to sustain differential disruption, regardless of impact magnitude as predicted by the Holborn postulate. Importantly, our findings also identify sulcal depths in addition to midline structures as sites of high strain concentration, reconciling prior finding of significant pathology in chronic traumatic encephalopathy, in both sulcal depths and midline structures (Alosco et al., 2020, Bieniek et al., 2021, Buckland et al., 2019, Chen et al., 2018, Barrio et al., 2015, Arena et al., 2020). We also find that both gray and white matter are under disproportionate strain concentration, reconciling prior evidence for both gray and white matter changes in mTBI patients (Hirad et al., 2019, Grossman et al., 2012, Palacios et al., 2020, Bazarian et al., 2012, Bazarian et al., 2014, Hellstrom et al., 2017, Lipton et al., 2009, Mayer et al., 2010, Burrowes et al., 2020, Ling et al., 2013, Ross et al., 2012, Little et al., 2010). In this regard, our findings underscore the importance of comprehensive assessment metrics that are sensitive to changes in both gray and white matter. In particular, the use of AFD is an important index that allows for *in vivo* quantification of both white and gray matter injury.

Finally, our normative MRE maps show that sulcal depths, midline callosal regions, and mesial temporal structures are exposed to relatively (relative to the rest of the brain) higher shear strain under low-amplitude mechanical loading. This suggests that so-called “non-concussive” impacts may still drive these mechanically vulnerable regions past an injurious strain threshold, without concomitant acute or subacute clinical syndromes. As such, our findings and approach provide a potential mechanistic insight into the sulcal and perivascular pathology observed in CTE. Future work combining subject-specific finite element models, head-impact kinematics, and longitudinal AFD measurements will be essential to estimate strain thresholds for injury and to define exposure–response functions for repetitive sub-concussive impacts.

## 5 Limitations

There are important limitations of our findings that motivate next steps. First, the magnitude of impacts during mild Traumatic Brain Injury (mTBI) substantially exceeds the amplitude of the non-injurious actuation used during Magnetic Resonance Elastography (MRE) strain measurements. Replicating concussive loading magnitudes in living humans is not feasible for ethical reasons and therefore, our study does not represent direct biomechanical causation of injury. Instead, we demonstrate a statistical correlation indicating that regions prone to higher shear strains under controlled MRE conditions align with areas commonly affected in mTBI patients. Nonetheless, our findings align with prior computational modeling studies of high-magnitude, impulsive head impacts, which also identify deep central brain structures, including the corpus callosum and gray-white matter interfaces, as regions of high shear strain concentration. We have addressed this by using separate cohorts of (acute, sub-acute, and chronic) mTBI patients to validate the premise that brain regions exhibiting differential strain concentration under conditions of non-injurious loading correspond to those observed in mTBI and studies involving gyrencephalic animals and cadavers subjected to higher impact magnitudes support the observed differential strain concentration and pathology in the same regions we report (Ommaya et al., 1994, Margulies et al., 1990, Smith et al., 1997, Arbogast and Margulies, 1998, Alshareef et al., 2020). The correspondence of strain concentration across low and high magnitudes, with controlled actuation of the human skull, may be partially explained by the stereotyped response frequency of the brain, irrespective of input frequency and magnitude (Alshareef et al., 2020, Alshareef et al., 2018). It is important to acknowledge that our strain metrics used here differ from axonal strain or white matter-specific strain measures used in some computational modeling studies, necessitating caution in direct comparisons. As such the discrepancy in loading magnitudes highlights an important gap in our understanding of how strain patterns under different loading conditions relate to injury outcomes. Future research would address this limitation by leveraging advanced brain finite element models to simulate and compare brain strain responses across a spectrum of external biomechanical loads, from non-injurious to injurious levels. These models have the potential to validate and extend the current findings by elucidating the biomechanical mechanisms underlying strain-dependent tissue vulnerability in mTBI and in those exposed to repetitive head impacts, thereby bridging the gap between experimental strain patterns and clinical injury pathology.

A second limitation is that our study is limited by the absence of information regarding the direction of impact in our concussion cohort. We have addressed this by focusing on regions that exhibit strain concentration irrespective of the direction of impact. Nonetheless, our findings compel a shift in practice, which is to prospectively assess the direction of impact in clinical concussion assessment, in support of future tests of how strain concentration in specific structures is modulated by the direction of force propagation.

A third limitation is that, although we emphasize patterns of high strain concentration that are invariant to frequency and direction of force loading, not all regions exhibit such invariance. Factors such as frequency and force direction undoubtedly play a role in determining strain patterns, as highlighted in the work of Laksari and colleagues (2018), where frequency-dependent dynamics were linked to amplified strain in specific regions (Laksari et al., 2020). This underscores the importance of future modeling efforts to account for individual patterns of head impacts and tissue disruption (Supplemental Fig. 2; see Laksari et al. for more details).

Fourth, in the acute sample, all controls were male whereas the mTBI group included both males and females. Although our sex-stratified analyses showed that high-strain AFD reductions persisted when restricting the comparison to males and were also present in acute females, the number of female participants was small, and we lacked female controls. As a result, we cannot fully characterize sex effects or sex-by-strain interactions in the acute phase, and future studies with larger, sex-balanced cohorts will be needed to test whether the strain–AFD relationship in mTBI in general differs by sex. Indeed this particular limitation and solutions thereof apply to age as well, as an important biologically salient variable in mTBI.

Fifth, OSS has not been as extensively used in the field as MPS and related metrics such as MPS rate. Previous computational modeling studies using these metrics have made significant contributions by establishing strain thresholds, distributions, and their relation to injury. While MPS captures both shear and longitudinal strains, our study focuses on testing Holbourn’s theory, which explicitly posits that shear strain, rather than longitudinal strain, plays the dominant role in TBI and dictates the resulting injury pattern. Therefore, we determined that OSS is more appropriate for our investigation. However, our decision to use OSS does not diminish the importance of longitudinal strain. Despite the high correlation between OSS and MPS (R^2^ = 0.83), it is reasonable to attribute at least some of the remaining 17% of variance to the contribution of longitudinal strain. Consequently, the role of MRE-measured MPS as a combined metric of shear and longitudinal strain to mTBI remains an important topic for future research. Future studies could directly compare OSS and MPS – ideally by computing both metrics from subject-specific FE models in the setting of repetitive head impacts and relating them to longitudinal AFD changes – to determine which strain measure (or combination thereof) best captures injurious mechanical loading.

While we demonstrate that differential energy arrival and reduced energy dispersion as driving mechanisms behind high strain concentration, it is important to note that other biomechanical factors such as distance from the center of rotation, axonal fiber orientation, strain rate, microstructural discontinuities, and material differences at gray and white matter interfaces have all been tested and directly implicated in driving injury and injury distribution in cadaveric, finite element, and animal studies. How these factors contribute to injury strain concentration measured with MRE would be considered in future work.

A final limitation concerns the heterogeneity of mTBI signs and symptoms. The framework we have articulated is largely based on the empirically established premise that there is a stereotyped pattern of injury in mTBI (Holbourn, 1943, Ommaya et al., 1994, Arbogast and Margulies, 1998, Graham et al., 1995, Ommaya, 1995). At the same time, there is a wide range of inter-individual variability across the acute, sub-acute, and chronic phases of post-concussive recovery in terms of the pattern of signs and symptoms (Chakroun-Walha et al., 2017, Buchanan et al., 2021, Elliott et al., 2021, Biagianti et al., 2020). Furthermore, there is a lack of sex balance in our acute mTBI group and their corresponding controls, which could not be addressed due to the use of a preexisting dataset. Future studies should aim to achieve a more balanced representation of sexes to enhance the generalizability of the findings.

## 6 Conclusion

Despite these limitations, our findings reinforce what is common across m/TBI (Ommaya et al., 1994, Cullen et al., 2016, Graham et al., 1995), in a way that is grounded in the biomechanics of shear wave propagation through tissue. The flip side of this is the exciting prospect that person-specific patterns of strain concentration may be inferred from individual variability in brain anatomy. That approach could move the field closer to developing biomechanically grounded prognostic models about where injury is predicted to differentially accumulate in a given individual for a given head impact. Future work can seek to relate how variability across mTBI patients in direction and force of skull loading, interacts with variability in viscoelastic properties of brain anatomy, to explain variability across patients in patterns of tissue injury and clinical signs and symptoms.

## 7 Transparency, rigor and reproducibility

Steps were taken to ensure the rigor, reproducibility, and transparency of the study. All MRI analyses were conducted to maintain independence between voxel definition and hypothesis testing, and regions of interest (ROIs) were based on a widely used atlas of codified anatomical regions to minimize bias. Data acquisition protocols were standardized and designed to be feasible for all participants. Quality control procedures, including the use of MRIQC and phantom-based checks, were implemented to ensure consistent MR signal quality over time. We used publicly available MRE data and will make the mTBI data available to other researchers upon request, contingent upon meeting the appropriate IRB requirements. Analyses employed widely validated OpenSource tools (e.g., FSL, ANTs, FreeSurfer, and Dipy), and any custom code used in the analysis is OpenSource and available on HiradLab GITHUB to promote transparency and reproducibility.

Although sex imbalance in the acute mTBI cohort was a limitation due to the use of preexisting datasets, future studies will aim for more balanced representation. All steps were documented to support reproducibility, and data from this study will be made available upon request in compliance with journal policies. Together, these measures ensure a high level of rigor and reproducibility in the presented findings.

## 8 Authorship confirmation/contribution statement

- Hirad (AH): Led Conception and Design, Analysis and Interpretation, Data Collection, Writing the Manuscript, Critical Revision.
- Mix (DM): Contributed to Analysis and Interpretation, Writing the Manuscript, Critical Revision.
- Venkataraman (VM): Contributed to Analysis and Interpretation, Data Collection, Critical Revision.
- Meyers (SM): Contributed to Analysis and Interpretation, Data Collection, Critical Revision, Approval of the Manuscript, and Agreement to be Accountable.
- Mahon (BMZ): Contributed to Conception and Design, Analysis and Interpretation, Writing the Manuscript, Critical Revision.

## 9 Funding disclosure

Preparation of this ms was supported, in part, by NIH Grants R01NS089069 and R01EY02853, and the Chuck Noll Foundation to BZM.

## CRediT authorship contribution statement

**Adnan A. Hirad:** . **Doran Mix:** Writing – review & editing, Writing – original draft, Investigation, Funding acquisition, Conceptualization. **Arun Venkataraman:** Writing – review & editing, Visualization, Software, Formal analysis, Conceptualization. **Steven P. Meyers:** Writing – review & editing, Resources, Investigation, Funding acquisition, Data curation, Conceptualization. **Bradford Z. Mahon:** Writing – review & editing, Writing – original draft, Resources, Investigation, Funding acquisition, Formal analysis, Conceptualization.

## Declaration of competing interest

The authors declare the following financial interests/personal relationships which may be considered as potential competing interests: AAH and BZM hold IP, predicting injury from mTBI using MRI (US12178591B2).

## Data Availability

All data needed to evaluate the conclusions in the paper are present in the paper and/or the Supplementary Materials. The deidentified and defaced data from mTBI cohorts are available upon request from the corresponding author. The MRE data is public data and described in ref 49.
