# Supplementary material for: Noninvasive mapping of shear strain predicts the anatomical distribution of mild traumatic brain injury
Source: Neuroimage Clin. 2026 Feb 19;49:103974. doi: 10.1016/j.nicl.2026.103974 (PMC12945589; doi:10.1016/j.nicl.2026.103974)
Supplement: Supplementary Data 1 [file mmc1.docx]

**Supplemental Online Materials**

**Title:** Noninvasive mapping of shear strain predicts the anatomical distribution of mild traumatic brain injury

**Author List**: Adnan A. Hirad^1-3^, Doran Mix^1, 4^, Arun Venkataraman^5^, Steven P. Meyers^6,7^, Bradford Z. Mahon^7,8,9^

**Affiliations:**

1. Department of Surgery, University of Rochester Medical Center, Rochester, NY, 1462, USA
2. Department of Neuroscience, University of Rochester Medical Center, Rochester, NY 14642, USA.
3. Del Monte Neuroscience Institute, University of Rochester, NY, USA
4. Department of Biomedical Engineering, University of Rochester Medical Center, Rochester, NY, 1462
5. Department of Physics and Astronomy, University of Rochester, NY, 14623, USA
6. Department of Imaging Sciences, University of Rochester Medical Center, Rochester, NY, 1462, USA
7. Department of Neurosurgery, University of Rochester Medical Center, Rochester, NY, 1462, USA
8. Department of Psychology, Carnegie Mellon University, Pittsburgh, PA 15206
9. Neuroscience Institute, Carnegie Mellon University, Pittsburgh, PA 15206

**Supplemental Table 1: White matter tracts under direction-of-head-impact invariant strain concentration**

| **White Matter Tracts** | **Numbers of Voxels (tscore > 4, Corrected p < 0.05)** |
| --- | --- |
| Anterior thalamic radiation L  Anterior thalamic radiation R | 40  16 |
| Corticospinal tract L  Corticospinal tract R | 268  343 |
| Cingulum (hippocampus) L  Cingulum (hippocampus) R | 189  240 |
| Forceps major (Splenium)  Forceps minor (Genu) | 882  91 |
| Inferior fronto-occipital fasciculus L  Inferior fronto-occipital fasciculus R | 607  604 |
| Inferior longitudinal fasciculus L  Inferior longitudinal fasciculus R | 1899  1187 |
| Superior longitudinal fasciculus L | 114 |
| Uncinate fasciculus L  Uncinate fasciculus R | 34  49 |

**Supplemental Figure 2:**

**
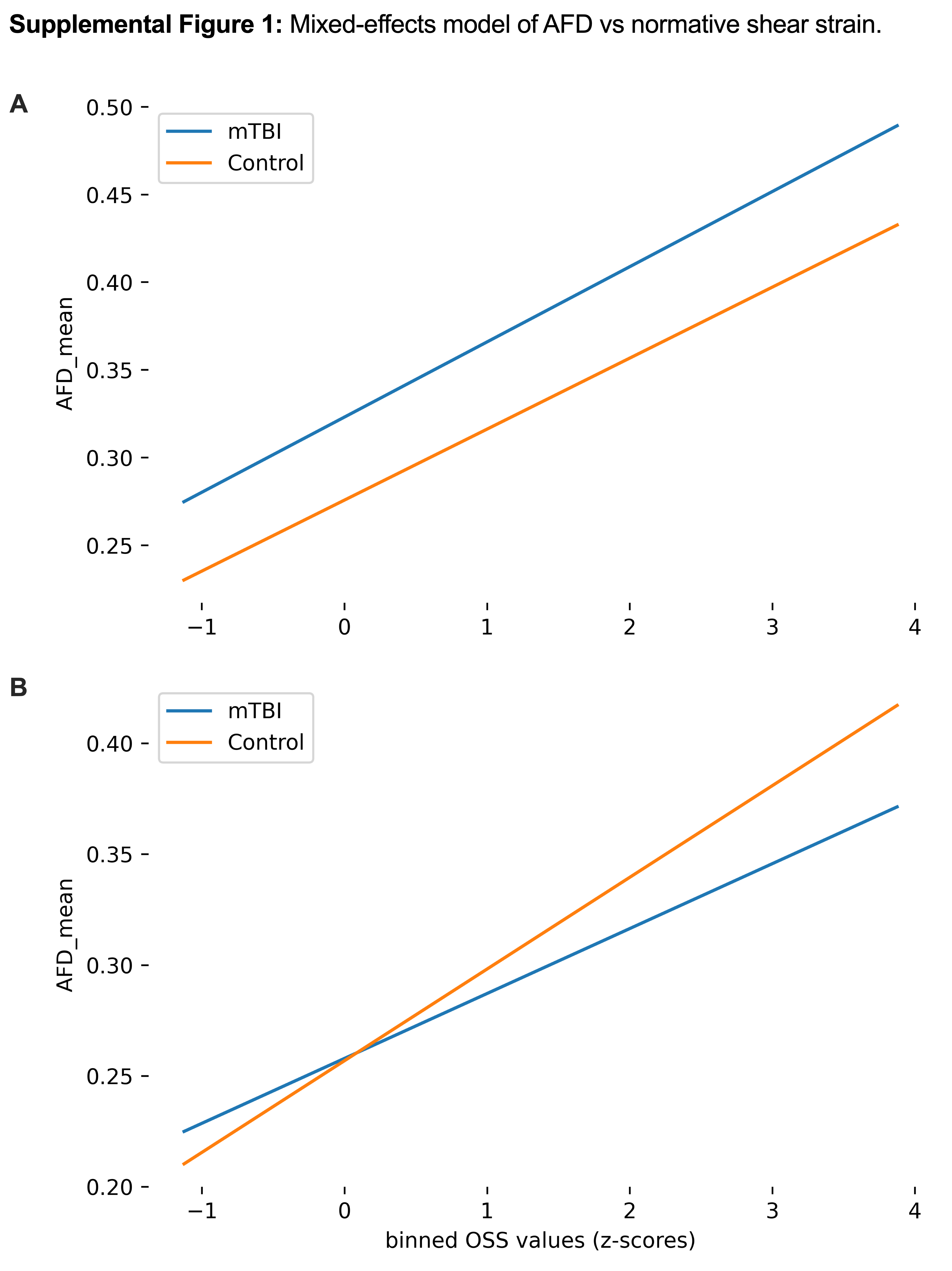
**


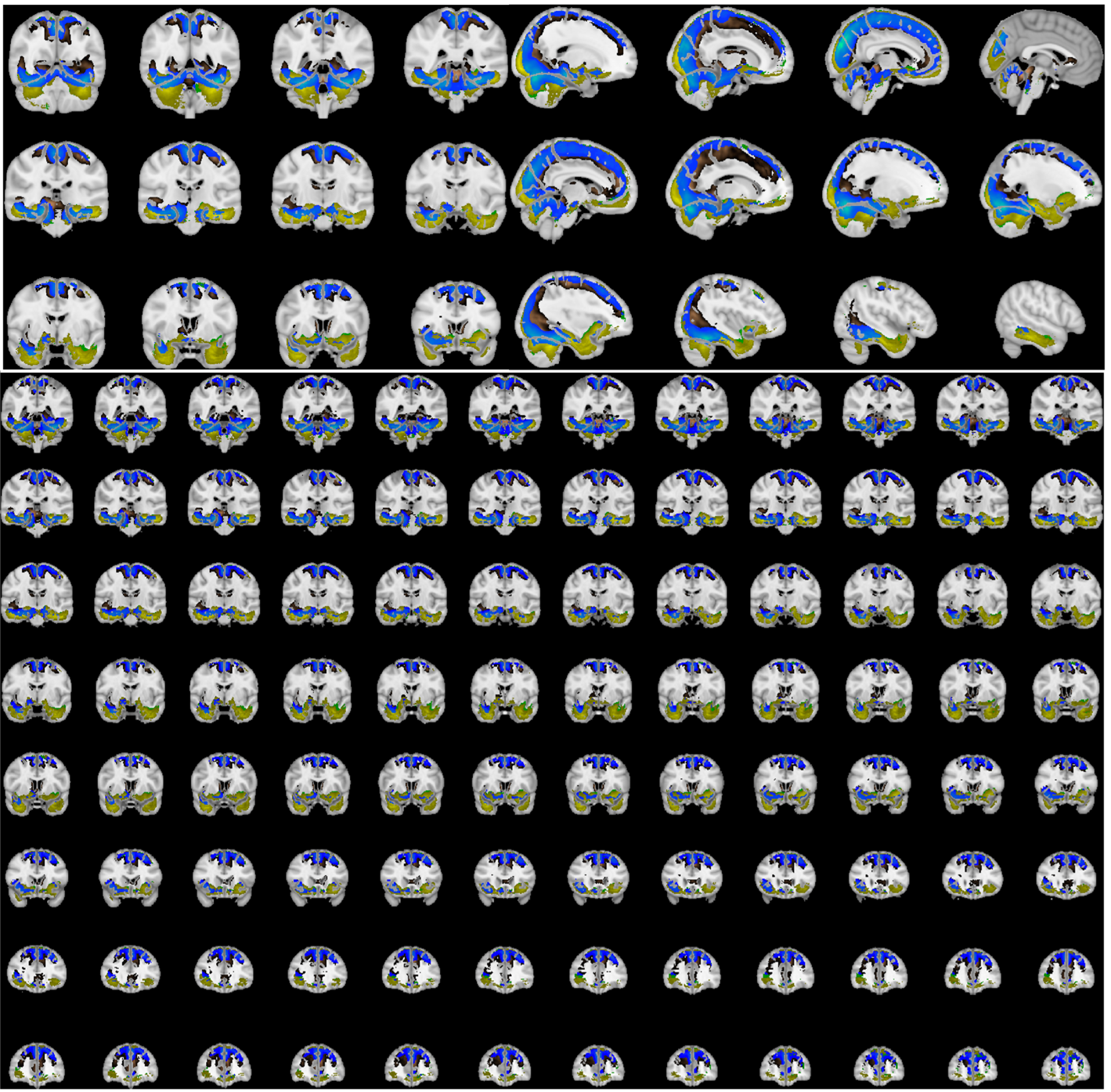
**Supplemental Figure 2:**
